# Integrating patient perspectives in medical decision-making: a qualitative interview study examining potentials within the rare disease information exchange process in practice

**DOI:** 10.1186/s12911-019-0911-z

**Published:** 2019-09-18

**Authors:** Ana Babac, Verena von Friedrichs, Svenja Litzkendorf, Jan Zeidler, Kathrin Damm, J.-Matthias Graf von der Schulenburg

**Affiliations:** Center of Health Economics Research Hannover (CHERH), Leibniz Universität Hanover, Hanover, Germany

**Keywords:** Shared decision-making, Rare diseases, Expert patient, Patient preferences, Patient centered care, Qualitative research

## Abstract

**Background:**

Many European countries have recently implemented national rare disease plans. Although the network is strengthening, especially on the macro and meso levels, patients still go a long way through healthcare systems, with many health professionals involved and scarce evidence to gather. Specifically, patient involvement in the form of shared decision-making can offer further potential to increase healthcare systems’ efficiency on a micro level. Therefore, we examine the implementation of the shared decision-making concept thus far, and explore whether efficiency potentials exist—which are particularly relevant within the rare disease field—and how they can be triggered.

**Methods:**

Our empirical evidence comes from 101 interviews conducted from March to September 2014 in Germany; 55 patients, 13 family members, and 33 health professionals participated in a qualitative interview study. Transcripts were analyzed using a directed qualitative content analysis.

**Results:**

The interviews indicate that the decision-making process is increasingly relevant in practice. In comparison, however, the shared decision-making agreement itself was rarely reported. A majority of interactions are dominated by individual, informed decision-making, followed by paternalistic approaches. The patient-physician relationship was characterized by a distorted trust-building process, which is affected by not only dependencies due to the diseases’ severity and chronic course, but an often-reported stigmatization of patients as stimulants. Moreover, participation was high due to a pronounced engagement of those affected, diminishing as patients’ strength vanish during their odyssey through health care systems. The particular roles of “expert patients” or “lay experts” in the rare disease field were revealed, with further potential in integrating the gathered information.

**Conclusions:**

The study reveals the named efficiency potentials, which are unique for rare diseases and make the further integration of shared decision-making very attractive, facilitating diagnostics and disease management. It is noteworthy that integrating shared decision-making in the rare disease field does not only require strengthening the position of patients but also that of physicians. Efforts can be made to further integrate the concept within political frameworks to trigger the identified potential and assess the health-economic impact.

## Background

### The relevance of shared decision-making and patient perspectives

Historically, a paternalistic decision model has been established within several healthcare systems [1, 2]. However, as healthcare systems shift toward patient-centered care, the patient’s role has become increasingly prominent. Integrating concepts of evidence-based medicine and patient perspectives, such as the inclusion of patient preferences, has also become increasingly relevant [3, 4].

The concept of shared decision-making (SDM) was first mentioned as such in 1982 [5]. It has been positioned as a centerpiece between paternalistic models, in which physicians dominate the decision-making process, and an informed patient choice model, in which the physician provides information but the patient assumes a leading role [4, 6]. The most often-cited concept originates from the work of Charles et al. [6], who defined SDM as a collaborative process between patient and provider based on a discussion of options, evidence, and potential benefits and harms; this especially considers the patient’s preferences and situations [6].

A review of literature published between 1996 and 2011 by Blanc et al. [7] identified 1285 out of 229,179 publications in 15 journals addressing the topic of SDM. In this context, it was identified that publications in medical journals increased exponentially during this period, which indicates the topic’s growing relevance. However, the meaning of SDM is often assumed rather than interpreted through SDM testing models [8]. Other studies suggest that existing SDM models only partially reflect the factors that influence patient empowerment or the breadth of their further potential [9, 10]. Literature suggests that SDM concepts augment patients’ satisfaction with the healthcare system, especially regarding the quality of care [12]. Further, this can also positively affect health outcomes. Patients’ poor compliance and inappropriate use of medicines arise from poor communication, the patient’s lack of understanding of how the drug is expected to work and its potential side effects, and a failure between the patient and physician to find a common ground or concordance [13, 14]. Desrroches et al. [4] suggest that SDM should be chosen as an ideal chronic disease strategy to improving compliance with medications and therapeutic processes, which is a major public health issue. This suggests a particular relevance regarding diseases with chronic life cycles.

Nevertheless, healthcare professionals consistently fail to facilitate patient involvement, and even fewer include patient preferences in care [15]. An appropriate level of SDM does occur in practice, although only in approximately 10% of cases [16], suggesting that although literature broadly covers the SDM process, further efforts are still necessary to expand its usage.

### The rare disease context

Estimations reveal that approximately 350 million people are affected by rare diseases worldwide. Many rare diseases can be traced to a genetic origin, often linked to a chronic course of diseases as well as severe symptoms [17]. Although various different health conditions affecting different parts of the human body are subsumed under the term “rare diseases,” these people share common difficulties [18].

Consequently, worldwide rare disease national plans have been implemented, with 20 implemented overall in Europe from 2004 to 2014. These policies focus on centers, networking, research, disease registries, coding, therapies, information provision, and patient organization [19]. In this regard, patients are solely and indirectly included through patient organizations. However, the German National Plan for cancerous diseases indicates that direct patient involvement is also strengthened through empathetic communication and including patients in decision-making, as noted in action field number 4 [20].

On the one hand, information needs and preferences have already been examined in the rare disease field [21–23]. However, concepts of shared decision-making have thus far only been evaluated in such contexts as diabetes [8], mental illnesses [9], coronary heart disease [24], and cancer screening programs [25]. Evaluations regarding rare diseases that include all people affected as well as physicians are still lacking.

On the other hand, physicians can provide extensive information on treatment options and managed care contracts in prominent disease areas, such as diabetes and cancer. However, information in the rare disease field is often scarce, and those affected often become “experts” of their own disease by capturing substantial information during their long odyssey through healthcare systems [26].

### The study’s aim

Triggering this information through effective information exchange strategies, such as shared decision-making, could contribute to the efficiency of, and overall satisfaction with, healthcare systems. Therefore, the purpose of the underlying study is to examine the implementation of the SDM concept in Germany. Further, the study explores whether efficiency potentials exist for the healthcare system within the rare disease field. The triangulation of patient interviews with family members’ and physicians’ opinions controls for the results’ validity and practicability. The results also contribute to the framework of shared decision-making, as this framework identifies potentials resulting from the specifics of rare conditions.

## Method

The study was based on Mayring’s qualitative research methodology [27]. A qualitative setting was chosen as the goal to observe a holistic picture of SDM, rather than analyze by focusing on the research topic’s provisional aspects [28]. The qualitative setting also allows participants to independently address important aspects. Therefore, the outcomes illustrate participants’ current, actual experiences, and are not channeled by interviewers. The material was collected through semi-structured interviews and evaluated based on a qualitative content analysis in an inductive-deductive approach. The research items in this context are chosen from existing concepts, and all evolving topics in the evaluation process are assigned to the research items and the evolving subcategories [27].

This study’s empirical evidence is derived from three data sets, offering information on three comparison groups. These sets were chosen by considering the information rendered on the research topic, and whether the sets contribute more to a rich description then one data set alone [29]. Further, comparisons within qualitative research add more to the full understanding of the issue than analyzing differences [30]. Hence, this study triangulates patients, or the directly involved; their relatives, or the indirectly involved; and physicians, as the counterparts within the decision-making process. Therefore, the comparison groups’ different perspectives could be carved out.

### Participant recruitment

Overall, a qualitative, non-random quota-sampling technique was used. In this context, the population is first determined regarding specific qualities, and certain quotas are then recruited from these subgroups [31].

Patients and family members were recruited by the Freiburg Centre for Rare Diseases at the Department of Dermatology of the University Medical Centre. As “rare diseases” summarizes many conditions with different appearances, the goal was equal coverage of the following disease areas (*n* = 11): skeletal dysplasia, neuromuscular disorders, immunodeficiencies, genetic eye disorders, genetic skin disorders, connective tissue disorders, genetic kidney diseases, cystic fibrosis and lung diseases, an inherent disturbance of hematopoiesis, inherent metabolic disturbances, and genetic diseases of the digestive tract. However, the interview results often indicated an overall complex, systematic involvement. We also strive for a balanced recruiting of female and male participants, as well as a participant from different age groups. We included at least nine patients with a long path to diagnosis, defined as lasting at least 10 years. The inclusion of relatives was necessary, as many rare diseases affect children, who are ineligible for interview, and participants needed to be at least 18 years old. Alternatively, a close relative was invited to answer the questions. Potential participants were chosen from the rare disease center’s clinical register and were contacted as part of a clinical visit with visiting patients randomly chosen. Patients did not agree to participation in advance. Further, all participants who signed an informed consent agreement and were assigned to an interviewer remained in the study.

The interviewed physicians are part of the field of action of people affected by rare diseases defined as such by Meuser and Nagel [32]. Physicians as health experts serve to supply information from the operating contexts of those affected, covering the overall spectrum of the providing health care structure [32]. Therefore, physicians were selected in accordance with their profession, including general practitioners, specialists, and clinicians. Moreover, guides in the rare disease field were also questioned. The term “clinicians” in Germany represents medical experts working in hospitals, while “specialists” operate in private practice. Guides differ in their qualifications and are equally trained to direct patients suffering from rare diseases, but were only included in instances in which a medical background could be determined. The Centre of Quality and Management in Health Care, in the State Medical Chamber of Lower Saxony in Hannover, was responsible for recruiting medical professionals. All physicians were recruited within the geographic region of Lower Saxony, representing both urban and rural areas in Germany. As clinical guides occur less frequently, this entire subgroup was recruited from all regions in Germany. The following criteria were employed to create appropriate subgroups: residence, such as rural, urban, or metropolitan; single versus group practice; medical care level, such as basic, regular, specialist, and maximum; and level of the physician’s medical experience, with hierarchy levels of assistant physician, senior physician, and chief physician. A physician’s hierarchy level is referred to as an indicator of the experience of hospital physicians. Therefore, different modes of employment, called “hierarchy levels” in this case, were differentiated. In German hospitals, three different professional positions are common, beginning with the lowest level of “assistant physician,” followed by “senior physician,” and then the most experienced level of “chief physician”. Therefore, the sample covers the heterogeneous area of healthcare provision relative to the research topic of interest, and thus, covers the field of action defined by Meuser and Nagel [32] regarding physicians as experts on rare diseases in Germany.

### Data collection

Three different interviewers queried patients and family members between March and November 2014, while two other interviewers met with physicians between April and October 2014.

Interviews were held face-to face (patients and relatives: 40; physicians: 26) and by telephone (patients and relatives: 29; physicians: 7). Not all interviews could be conducted face-to-face due to appointments on short notice, the need for extensive disproportionate travel, or participants’ preference.

Interviews were conducted according to a semi-structured interview guide. The interview guide was developed during a mutual workshop of the research group led by an experienced external qualitative researcher. The qualitative researcher presented the relevant qualitative research guidelines. Afterwards, the research group presented the potential interview questions. During our mutual discussion, questions were adapted further, as the first draft included questions that did not induce narration appropriately. The second drafts of the question and sub-question sets containing further minor wording adaptions was sent to the research group via email to reach a final consensus on the question set. The interview flow was initiated through a narrative question requesting rare disease experiences from the onset. Sub-questions concerned the diagnosis, and therapy and disease management were only posed when questions were not autonomously addressed. This approach was chosen to identify emerging SDM aspects, rather than proffering the SDM concept for the participants. Thus, we strove to avoid overestimating SDM effects, which can occur due to expressive reporting, when directly inquiring about a concept. Further, requests were made as interviewers observed the necessity. Questions on information-gathering behavior and interchanges were subsequently posed.

The interview study was embedded into the creation of a national rare disease Internet platform in Germany (Zentrales Informationsportal über seltene Erkrankungen, or “ZIPSE”), which enables patients and family members to actively gather quality-assured evidence [33].

The interview guide was pretested with one patient and one family member, and was then adapted to include the perspectives of participants who have experienced their diseases since birth. The following interviews appropriately covered the different courses of rare diseases, and therefore, this study could optimally cover the different paths through the healthcare system and interactions with healthcare specialists could be ideally covered. The physician’s interview guide was accordingly developed to align the guide’s structure, and was pretested by interviewing one physician (female, 43 years). Piloting the interview guide demonstrated that relevant cases could be appropriately triggered based on the interview guide. Nevertheless, some adaptations were necessary, as the healthcare professionals offered different perspectives on the topic. The research group mutually reviewed both interview guides to align standardized procedures. We first conducted a trial run of an interview to practice the procedure and control for the fit of the posed interview questions. During the following interviews, participants’ pathways through the healthcare system, also referring to interactions with the contacted medical professionals, diagnosis, and potential therapies, as well as the diverging information sources and/or information access points, were well described. Qualitative research also gives the option to further adapt the interview guide along the way. Therefore, we continuously checked for further adaptation needs. However, due to in-depth narration of interviewees and coverage of the addressed topics, no further adaptations were deemed necessary.

### Data analysis

All participant interviews were audio recorded, and later transcribed with the aid of F4 transcription software (Version 6, Dr. Dresing & Pehl GmbH in Germany). A standardized interview protocol was also distributed to interviewers of patients and family members to document any special circumstances potentially relevant in interpreting the collected data. A standardized transcription booklet was developed for patients, relatives, and physicians, and was used as a transcription guideline. The transcription booklet offers a standardized definition of different transcription strategies and codes, where diverging options were possible. This defined the anonymization of participants and locations, as well as the handling of any incomprehensible audio sections.

All steps of a qualitative content analysis were then recognized [34]. Hence, transcripts were analyzed based on a directed qualitative content analysis [27]. An inductive-deductive approach was used. Predefined items were identified in a deductive first step using Charles et al.’s [6] predominately used definition of SDM. Therefore, the following items were noted: 1. The “patient-physician relationship,” 2. “Participation,” 3. “Information exchange,” and 4. “Decision-making.” Second, subcategories were developed, assigned and revised in a stepwise procedure following Mayring’s [27] inductive category development process. Finally, two further researchers revised the evolving items to ensure both formative and summative reliability and any differences were addressed and included in the data analyses. Ultimately, the patients’, family members’, and physicians’ results were triangulated. Evolving items from physicians’ and relatives’ interviews were matched with already identified items from patients’ interviews, where appropriate. Otherwise, a new subcategory was deemed necessary. All quotations were reviewed by a native speaker.

## Results

Appendix 1 displays detailed characteristics for the participating patients, family members, and physicians. Overall, 55 patients, 13 family members, and 33 physicians were interviewed as a part of the study, although at least one participant could not independently participate in the interview study due to the severity of their illness. The patients’ median age was 52, family members was 44, and physicians was 48; 67% of patients, 61% of family members, and 30% of the physician subgroup were female. The diseases’ severity were self-assessed, with 9% of patients rating their disease severity as mild, 47% as medium, and 38% as severe. 6% did not state any specifications.

The interview results were marked with a code consisting of an interview number and letter: “P” represents the patient, “A” represents an “acquaintance or family member,” “GP represents the general practitioner,” “CP” represents clinical physicians, and “EP” represents expert physicians. The following themes regarding experiences, decision-making processes, and information exchange procedures were identified in the rare disease field:

### Item 1: the relationship between patient and physician

#### Nurturing trust-building processes

Patients report that trust is an important basis for effective communications about their disease. If a relationship of trust has not evolved, patients will prefer to make their decisions and search for the answers to their health questions on their own, often through the Internet (P37). Communication can lay the foundation for a trusting relationship. For example, one physician reported that she would not write reports, but trusted the patient’s understanding of their rare disease (GP02).

Moreover, a trusting relationship is nurtured when physicians admit the limits of their knowledge of rare diseases and make the appropriate contacts to compensate for this gap (GP04, GP08). A trusting relationship is particularly strong when patients can obtain answers to current health questions—no matter how straightforward—and when physicians take the time to research and discuss these questions with their patients (P06).

Several aspects within the patient-physician interaction must be considered to avoid mistrust. Our patient respondents were disappointed with highly qualified physicians who lack information about rare diseases (P05, P08, P27). Patients also believed that physicians could hardly empathize with their conditions (P22), and searched for medical professionals who cared (P27). This, and the lack of a constant contact person, especially in outpatient care, led to high dissatisfaction and patient mistrust. Alternatively, patients positively perceived the existence of a permanent contact person (P01, P24). Further, a relationship of trust is undermined by misdiagnoses (P05, P17), as patients recognize that physicians experience difficulty in diagnosing rare diseases. Although they somewhat understand physicians’ struggle, patients also reported the need to search for a physician capable of diagnosing their disease (P34). Moreover, disappointment occurs when treatment is denied as a result of a high-risk disease classification due to comorbidities or age (P06, P38). Family members also report that they need to verify physicians’ information regarding treatment considerations, as therapy options are often scarce or newly developed, and they rather feel like “guinea pigs,” which feeds their distrust (A09). It is also reported that no elucidation by the physician occurred, although the diagnoses were already established, which increased suspicion (A10). The next step may involve a further increase in mistrust if patients—or family members, in this case—do not acquiesce to the opinion offered during the medical consultation (A05).

#### Dependencies

On the one hand, rare diseases often exhibit a chronic course and the relationship between patients and physicians is often characterized by dependence, which also highlights the importance of a positively perceived relationship. On the other hand, negative perceptions can lead to a change of physician. Those affected can organize in patient networks or self-help groups, which help them feel supported; therefore, they become empowered and can assume more responsibility for their disease management (P30).

Family members also report that one must be “lucky” to find an appropriate physician (A04), or that one “relies on a physician for better or worse” (A02).

#### The psychosomatic corner

It was found that physicians complain about patients who return with the same problems. As rare diseases are difficult to diagnose, patients suffering from rare diseases on the long path toward diagnosis may be categorized as psychosomatics, simulants, or hypochondriacs, or suffering from psychological problems, which further undermines a positive patient-physician relationship (A06, P08, P16, P22, P27, P28, P39, P49, P52, P56). Consequently, patients do not believe they are taken seriously, which can also lead to them terminating their treatment (A06, P12). Physicians also reflected upon the difficulty in diagnosing rare diseases and the problem of over-reported symptoms due to Internet information (CP01, CP05, EP02); however, these areas of interest are not linked.

#### Other participants

Patients also consider the recommendations of others affected through rare disease self-help groups. Other physicians are also predominantly involved, as diagnostic procedures may span a longer time period, and many different fields of expertise may be involved due to poly-systemic patterns. Patients even noted that they are glad when physicians cooperate with the resulting “referral marathon” (P13).

However, many patients reported their struggles when shifting from one physician to the next. This is especially the case when crossing the boundary from the stationary to the ambulatory healthcare sector, in which patients are confronted with a loss of information or a lack of information transfer, as it is assumed that other physicians are responsible for communication, or that this communication has already occurred in other events (A07, A08, P05, P13, P14, P18, P24, P26, P27, P28).

The following Table 1 summarizes the findings for Item 1 and provides corresponding anchor examples:

**Table 1:** Anchor examples for the “relationship between the patient and physician”

Identified Items	Anchor Examples
Nurturing trust-building processes	*“[...] But [regarding] the counseling, people often ask, ‘What do you say about that? What should I do? Should I really choose a hearth catheter, or should I drop it? What do you say about that?’” [Interviewer: ‘Hm.’] “And when I say, ‘Yes, go!’ or when I say, ‘No, don’t go!’—” [Interviewer: Hm] “That’s absolute. That’s what I experience again and again. They confide very much in our opinion. And when we endorse something, then it’s okay, and if we do not, then it’s not.” (Primary physician, female, 47 years old, GP03)* *“[...] As noted, one has no chance with physicians with such a disease. […] There are rare diseases, that’s disastrous. And physicians get a chance, somehow, to search for anomalies, to get clues about which diseases can be considered. In my case, it was rather stupid, as liver values were so much in the foreground; however, one only needed to regard the thrombocyte values. I don’t know how this can be done in an intelligent way, as based on this or that, it can be that. But private physicians in particular have a hard time identifying a proper diagnosis. And many people do not have the energy to transfer from one doctor to the next, as I do. Yes, that’s what one does.” (Patient, male, P34)*
Dependencies	*“[...] In the new city I live in, I have gone to hematologists, with whom I have not gotten along with at all, and the personal contact within the network has encouraged me to simply say, ‘No, I have a chronic disease and I am relying on that physician; if I do not get along with him, I need to change the physician.’” (Patient, female, P30)*
The psychosomatic corner	*“[…] And many physicians are still of the opinion that if there are no identifiable causes, then it is psychological. Then there are many dystonia patients who need to fight [the opinion] that this is simply not psychological, but neurological.” (Patient, female, P39)*
Other participants involved	*“I am lucky to have physicians who play along with this ‘referral marathon.’” (Patient, female, P13)*

### Item 2: participation during the decision-making process

#### Physicians’ commitment

Participation is perceived as minimal when no time seemingly exists to build an in-depth, trust-based patient-physician-relationship (P06). Patients were especially disappointed when physicians did not demonstrate engagement during the particularly long diagnosis process, with successive symptoms in the rare disease field (P19, P56). This was interpreted as a lack of effort to link symptoms in a networking approach (P06) and a lack of interest to further analyze the diagnosed disease (P50).

Alternatively, physicians expressed anger in not considering a rare disease (GP03). Further, a diagnosis is critical for patients, and especially when they experience a long path from their first symptoms to diagnosis. In this case, the patients strive to self-diagnose, for example, by making their own appointments for a verifying biopsy (P49). This importance is highlighted by their describing the difficulty in arguing for an understanding of their special physical needs due to their symptoms in front of family, friends, and colleagues with no diagnosis to build upon (P56).

Similarly, family members report that they even feel neglected by their pediatricians (A03). Patients’ reports also reveal that physicians must invest substantial time to work up the course of rare diseases, as patients experience a long path to diagnosis (P07). Physician respondents reported that cases take several weeks to work up (GP01).

The physicians also stated that the diagnosis does include a naming of the disease, but no scope, leading to limited decision-making (GP07). In this regard, they would like to offer more, but are bound to offer less due to limited therapy options or missing curative therapies within the rare disease field (P14).

#### Patients’ commitment

A particularly active form of engagement can be observed in family members, who described driving 500 km to search for a well-trained pediatric orthopedist or a specialized center for rare diseases (A01, A02, A06).

Physicians also highlight the particular engagement of family members, who urge proper diagnoses with the help of Internet information (A01, CP06, EP07). In this regard, physicians also emphasize the importance of engaged patients, as physicians need their participation to obtain anamnesis data they could not otherwise obtain (EP04).

Alternatively, patients also describe a passive form of interaction, in that they trust physicians’ expert knowledge and hope that they will share all relevant information. This subsequent form of interaction is a “supervisory” relationship (P55). Physicians also report that patients who are unwilling to cooperate (GP05) as well as those who are willing to co-operate especially experience high psychological strain, which they want to ameliorate or share (GP03).

The following Table 2 summarizes the findings for Item 2 and provides corresponding anchor examples:

**Table 2:** Anchor examples of “participation during the decision-making process”

Identified Items	Anchor Examples
Physicians’ commitment	*“Yes, […] I think that is the interesting part of the issue. Basically, the point is, they do not take into account this hypothesis. Simply, they always think about the obvious, at present, or what that could be. And basically, ‘It is not even a complex disease, and […] not even complicated to diagnose,’ [and they think,] ‘Oh, I can also add something about the diagnosis later on.’ But, one also needs to come up with it first. And there is the statement of the physician, whom I told that I suspected I have achalasia, and who then said, ‘Oh, that’s so rare; that’s not what you have for sure.’ They do not search for this.” (Patient, female, P56)*
	*“[...] And somehow, one has a contact person, and I have the feeling, and the neurologist says, ‘It is good that you take Valacliclovir, and I can also prescribe you physiotherapy. There is nothing more I can do.’ And there is this [feeling that] I would like to do more.” (Patient, female, P14)*
Patients’ commitment	*“So, in the run-up, a catastrophe [occurs] because one really has nobody [without a] diagnosis, [and it is] extremely difficult to somehow find the right doctor. Actually, there is, or there was at that point, as we started searching, […] no such centers for rare diseases that were developed during the last few years. And therefore, I should say, one naturally depends on the pediatrician in the first line, and one has to simply, that’s what we felt, have luck to get to the right physician.” (Family member, male, A06)*
	*“[…] Shingles, send a picture, then you know what it is. But when it’s something rare: no chance. But, you need to talk to the patient, you need anamnesis data.” (EP04)*

### Item 3: information exchange

#### Professional health knowledge on rare diseases

Patients appraise broad rare disease knowledge, which is interconnected with the trust-building process (P05). Patients also report satisfaction with a single contact point when expert information is attained (P02). However, patients must independently search for rare disease health experts, as health information on rare diseases is perceived as generally scarce (A08, A11, GP01, P17, P34, P38). Patients repeatedly reported that they are lucky to have found someone on short notice who can diagnose their disease (P47). Patients also felt uncomfortable when realizing that physicians need to research their sometimes incredibly rare disease (P49). Physicians noted that comprehensive guidelines augment this dilemma by making it difficult for patients to fall within general medical guidelines (EP03). Problems arise when a lack of information leads to misinformation that must be clarified by other health experts (P05). However, this process can be positively transformed by not only explaining the knowledge gap in rare diseases, but also transferring the case to experts on the disease (P55). Relative to health information needs, patients request information on innovative health procedures, for example (A08). When patient questions regarding their diagnosis remain unanswered, this feeds dissatisfaction and mistrust (P15, P16, P28). Patients are sometimes offered initial information, such as a diagnosis, and are subsequently asked to proceed by themselves to the next steps, such as finding information on the disease and searching for a proper physician (P08). However, physicians also highlighted the limitations of Internet information, and especially emphasized that physicians must take patients in hand when thoroughly structuring and sorting the gathered information (EP09). While physicians offer only indication names, self-help groups can render further in-depth information (A02). Moreover, patients acknowledge that it is impossible for physicians to grasp the entire spectrum of a rare disease’s effects, which are often linked to genetic mutations (P49).

#### Health information scope: between feeding fear and effective health management

Patients reported ambiguity about the scope of health information communications; on the one hand, they demand more information on diagnoses and diseases’ possible courses (P12, P24). On the other hand, some patients hold back information crucial for diagnosis due to a sense of shame (GP03). A diagnosis without further information exchange triggers a process of concern or dissatisfaction within patients (A10, P12, P24, P32). Some patients reported struggling with a language barrier resulting from the extensive usage of professional jargon, and needed more simplified information (P12). Patients reported feeling like “guinea pigs” when information was not rendered to a sufficient extent (P29). Presumably, patients’ parents in particular tend to extensively worry. In this regard, information should be communicated by keeping the patient and family members grounded and explicitly integrating reassurance as an instrument (GP09). Other physicians complained about a communication filter system, in that a questionnaire is first rendered, a doctor’s assistant reviews the notes and the physician himself is finally consulted (EP02).

#### The “expert patient”

Patients described themselves as deliverers of information on rare diseases, and they close existing knowledge gaps through the use of Internet information, for example (A03, P21). Younger physicians (KA04, KA06) in particular wished to individually inform patients through, for example, web-based information sources, but were concerned with other physicians’ negative reactions (P08, P30). In this regard, patients even described themselves as “good” patients when blindly trusting their physician (P12). Alternatively, physicians also reacted positively toward individuals’ searches for health information through web-based sources (EP01, EP11, KA07). Although those affected are aware of the limited quality of Internet information, such as the broad spectrum of users in web-based forums (A13), physicians perceive themselves as necessary information filters (EP09). Moreover, some health professionals discourage patients to search for web-based health information, as it is difficult to properly classify such information, leading to increased concerns (EP03, KA01, KA05, KA09). Physicians were concerned that the false alarms caused by misconceptions from Internet information could hinder the resources within healthcare systems that different positions urgently require (GP04). Other physicians differentiated between handling anxious patients, and discouraged information searches, and other patients, who are allowed to search for Internet health information (EP11).

Patients described a knowledge-gathering process in which they “hop” from one physician to the next and gather information from a primary information access point: the self-help group (A01, A02). Further information sources with growing importance include the expert centers for rare diseases, developed at university clinics throughout Germany. Those affected describe the knowledge transportation process from the center to the local consultant, in which the consultant takes the leading role by communicating the next steps in the medical process (A13). Patients’ high responsibility regarding their medical information and the search for physicians can be interpreted as a hurdle on the path to SDM. Further, patients may feel left to their own fate rather than achieving mutual health goals. As these centers increase in number, this step is also increasing in importance, and physicians discourage the use of forums due to the doubtful quality of their health information (GP03, EP03).

Information delivery is demanded at an earlier stage, in this case immediately at diagnosis (P31). In this regard, patients also remarked that they needed to urge physicians toward information generation, emphasized that these health experts are likely to forget that each patient must be informed from the onset, and demanded different scopes of information (P34).

Alternatively, patients also reported that those affected needed to inform physicians about their disease when they changed their permanent location and searched for a proper specialist (A06). Physicians complained that the reporting obligation does not exist due to a lack of obligatory transfers, resulting in the need to ask patients for their documents or call the prior provider (CP05, GP01).

Other patients noted that they communicated with physicians at the same level, avoiding unbalanced communication (P03). Patients suffering from rare diseases even urged physicians for a diagnosis, as their symptoms are often genetic. However, they were also thwarted by the physician, who stated, “How can you take upon yourself the right to intervene with the medical decision-making process? (P09)” Patients also tended to transfer on their own, by taking responsibility for their own disease management (P17).

Patients are called health “experts” on their own diseases, as they have unique knowledge on their symptoms and can gather information on their individual form of the disease (CP07). Similarly, family members are also called “lay experts,” as they also extensively gather and exchange health information (GP09).

The following Table 3 summarizes the findings for Item 3 and provides corresponding anchor examples:

**Table 3:** Anchor examples for the “information exchange”

Identified Items	Anchor Examples
Professional health knowledge on rare diseases	*“[...] Yes, I think that if I had the right diagnosis—if I had MS, for example, which was never really excluded—but if I had this as a diagnosis, then I could have told every physician, ‘Look, I’ve got MS.’ Then, everybody would know what that is, everybody would know what kind of constraints I have, and one would eventually show a little consideration for me.” (Patient, female, P16)*
Health information scope: Between feeding fear and effective health management	*“First, to protect the patient from himself, as the induced therapy wave or perhaps also false/ or diagnostic wave can also be harmful. But I also see it as a question of capacity of our health care system. That we are not able to smooth every false alarm induced by “chatrooms” through profound information coming from physicians.” (GP04)*
The “expert patient”	*“Especially those exchange websites. [I1: Um]. That he comes to me, and then somehow has enormous expectations and wants to tell me how it needs to be done [or not done], that’s difficult for me; but he can be right. Thus, I mean, who is the specialist for these diseases? Actually it’s the person afflicted. ‘Well, he’s got the symptoms, he knows how it was diagnosed, and he also knows what works for him.’ The real specialist on the disease is in general the sick person. When it comes to common diseases, we are also experts, because we experience them so often. When it comes to rare diseases—well, I think if the physicians were honest, they are sometimes just helpless, because, they just do not have it that often.” (Physician, female, 42 years, KA07)*

### Item 4: decision-making and agreement

#### Paternalistic communication

Paternalistic decision-making is unavoidable, and especially in emergencies (P17); transfers are also suggested in a paternalistic way (P06). Further, patients under diagnosis may not be asked whether they wish to be informed (P11, P28), and physicians may define the specifics of therapeutic interventions, and especially dosages (P22). Patients and family members described physicians who wished to convince them of a specific therapy option, and urged them toward a specific outcome, such as surgery (A05, P21). However, patients searched for a second opinion in severe interventions, ruling out alternative therapy options. (P51).

Some patients reported perceiving a paternalistic communication as positive due to the perception of protection (A09).

One patient opposed shared decision-making as well as the associated discussions, and noted that he needs a paternalistic communication approach, as this offers him reassurance (P17). Patients sometimes even make demands of their physicians by telling them what to do (GP03). Family members also highlighted the importance of their expectations of physicians’ alleviating their worries (A08), which physicians also expressed; the latter reported that family members tended to turn to physicians more quickly and frequently, and channeled parts of the responsibility toward their children (CP08). Physicians also highlighted their own responsibility when defining the diagnosis as a process of weighing the correct balance between clinical necessity and the willingness to be confronted with the disease, and especially in the case of parents (CP08).

Patients also described patients’ suppression mechanisms in coping with their disease, which suggest the necessity of a watchful physician (P47). A paternalistic attitude is also associated with protection, and offers hope by guiding patients in steps through a previously unknown health condition (P09). Patients also expressed the need for a general physician to lead them through the course of the disease (P11). Specifically, elderly patients tended to fully rely on the physician’s information, and reconciled themselves to their status rather than pushing for further health improvement (P48).

#### Informed (individual) decision-making

Informed decision-making is directly linked to the expert knowledge of many patients and family members, drawing many parallels. Patients reported that they feel as though they have no right to participate in the decision-making process in that the physicians focus on their diseases, but they would prefer to be holistically perceived as a person. Individual ideas are unwelcome, but perceived as questioning the physician’s expertise or authority (A12, P16). Similarly, physicians noted that individual information searches may lead to conflicts within the decision-making process between patients’ expectations and economic and medical action strategies (CP03, EP01).

However, patients reported that they solely discuss acute symptoms with their physicians. One case also noted that physicians were only consulted for general medical advice, as knowledge on this incredibly rare disease is so scarce that they preferred to consult other affected families (A12). Moreover, patients and family members also decide upon the point at which they consult a doctor, or whether they preferred to consider a second opinion (A12, GP04, GP05, GP06, EP07, GP09, CP02). Patients often manage their own basic daily care for these diseases, as well as preventive measures, such as sports and healthy nutrition (P54). Specifically, the patients themselves attend to their own daily chronic disease symptoms or discuss them in self-help groups (A03, P37).

However, some physicians also support patients’ informed individual decision-making processes by welcoming their self-reliance (P11, GP03), and encourage laying out the patients’ treatment choices (P10).

Some physicians report worry as a result of patients’ individual decisions. For example, if the physician decides upon a therapy, the patient may refuse to take the prescribed medication. The patient may oppose it due a lack of readiness for a radical procedure and its associated side effects (P28). A similar pattern occurs when patients are not involved in the decision-making process, and consequently, patients or family members often switch to a physician who more highly appreciates their opinions (A01).

Further, patients often report a struggle with the physician, and especially when they feel something is wrong but the physician cannot determine a diagnosis (P17, P28) or patients would prefer a specific therapeutic service (P38). Conflict also occurs when the physician urges health services that the patient opposes (P38). In contrast, physicians also struggle with patients who they describe as “overly engaged,” in that some patients attempt to pin down a diagnosis the physician does not endorse (GP03, GP09). In this case, decision-making in the rare disease field can play a specific role when there is no initial decision to make due to missing therapy options (P26). Another physician also described his struggle with solely servicing patient wishes, although he prefers to retain primary control of diagnoses (GP09).

#### Shared decision-making

The SDM concept was rarely described across all interviews, suggesting that it has not yet integrated into common healthcare practice—or at least in the rare disease field.

One patient emphasized the trust-based relationship with her physician achieved through the SDM process. Although she initially denied therapy, the application of SDM nurtured a trustful relationship, in that she finally admitted that if the physician determines she needs therapy, she will cooperate (P28).

Similarly, physicians can offer different therapeutic options but leave the final treatment choice to the patient (P24).

On the one hand, another positive effect of shared decision-making is that it can lead to the patient’s self-responsible understanding of their own disease and adherence to existing therapy options (P33). On the other hand, interviews confirm that although people affected with rare diseases depend on their physician’s expert knowledge, ignoring patient preferences can still lead to a change in physician (A03). For example, decisions can be made during a consultation that the patient does not agree with (P38), the patient and physician can disagree with diagnostic procedures (P39), or a specific therapy may not be prescribed (P30, P34).

Physicians admit that shared decision-making’s role is increasing, as patients want their own perspectives to be considered (EP04). Shared decisions’ importance has also evolved, as diagnoses can be made after a longer period of subtle symptoms. In this case, the disease has already lead to irreversible, adverse effects that patients must cope with as they struggle with whether the physician should have intervened at an earlier point in time (P49). The physician’s learning of the patient’s perspective and their medical and family history can lead to a better understanding of the disease (P22, EP08).

The following Table 4 summarizes the findings for Item 4 and provides corresponding anchor examples:

**Table 4:** Anchor examples for “decision-making and agreement”

Identified Items	Anchor Examples
Paternalistic communication	*“He knew [the disease], but I think […] he wanted to protect me. I had this feeling. He said, ‘Okay, we will first look at this.’ So first of all, [he] very slowly introduced the disease, and I had that feeling. And there was no malevolence, rather the contrary, he did know the disease very well, I have to admit. As I said, I cannot say ‘I think,’ but rather protection, so he rather wanted to protect the parents. No pessimism with such a disease and no giving up of hope, but rather, he said, ‘Let’s first of all wait and to the contrary care for it. First of all, you move on with your life as it is, you keep working and everything, not giving up anything.’” (Family member, female, A09)*
Informed (individual) decision-making	*“Therefore, I actually see my task in keeping the strings together during a transfer on my own, and I’d like to be invited to fill out one or another transfer form from somebody with a lot of knowledge. But to just nod something through in retrospect, that I have some reluctance with.” (Physician, male, GP09)*
Shared decision-making	*“[…] It really helped me, and if I listened to my physician, I would have taken Hydrea since 2009. These are chemo tablets, which have a lot of side effects. Where one asks himself or herself, ‘What is really the benefit? And what’s actually the best way to go?’ And that’s what Professor [NAME] does and that’s what he confirmed as unambiguous: that I can […] decide as a patient on my own as well. That I feel it on my own, and that I rather know what’s good for me. But that does not mean that he only speaks according to his audience, or that he tells me only what he thinks I want to hear. On the contrary, […] I can say that after this conversation I have gotten so far to say, if Professor [NAME] says that it’s time for therapy, […] it’s time for therapy.” (Patient, female, P28)*

## Discussion

### Summary of findings

The underlying interview study revealed that medical decision-making as a part of the patient-physician interaction is particularly relevant in the rare disease field due to a many medical contacts and a high dependency on the exchange and physicians’ engagement in general. All parts of the SDM process, as systematically added to existing literature using Charles et al.’s [6] framework, were indicated as increasingly relevant within the rare disease field in practice. However, the status quo demonstrates that the SDM agreement itself was rarely depicted or respectively perceived. In summary, the patient-physician encounter was characterized by a balancing of trust and mistrust and high dependencies, including an often-reported stigmatization of patients as stimulants and many participants were involved. Commitment was high due to a pronounced engagement of those affected. Within the information communication process, the particular roles of “expert patients” or “lay experts” in the rare disease field came forth; physicians’ and patients’ perspectives were triangulated to validate these findings, and all items were verified in their importance.

### Findings in the context of literature

First, this study analyzed the “relationship between the patient and physician” in Charles et al.’s [6] shared decision-making concept. The analysis revealed an imbalance between trust and mistrust in the rare disease field, whereby a trusting patient-physician relationship is a prerequisite for effective communication. Georgopoulou, Prothero, and D’Cruz [35], Dowell et al. [13], and the Royal Pharmaceutical Society of Great Britain, Merck, Sharp, and Dohme [14] all link a trusting relationship with a physician to many positive healthcare outcomes. Von der Lippe, Diesen, and Feragen [36] further deepen this analysis by describing a mistrust of doctors, and linking this to patients’ emotional reactions. Further, Ernstmann et al. [37] found a positive relationship between trust and patient enablement, validating our overall finding that shared decision-making is still infrequently used in practice in the rare disease field.

The interviews revealed that a trusting relationship can be nurtured by, on the one hand, medical experts’ transparent communication of rare disease knowledge, and on the other hand, their own engagement in the form of transfers or time invested. Literature notes transparent communication as one potential option among other tools, such as technical and interpersonal competence, physician agency, physician control, confidentiality, open communication, and disclosure [38] by systemizing the narrated items.

Aspects that undermine a trusting relationship and should be avoided include misdiagnoses, a lack of empathy, the lack of a constant contact person, the denying of treatments, and the “guinea pig” role in patients’ disease treatment when therapy options are scarce. These findings can shift the awareness to rare diseases that lack approved medications [39].

Further many patients report being placed in the “psychosomatic corner”, which hinders effective disease management. The acknowledgement of symptoms feeds into the patient-physician relationship Patients often report depending on a good physician, which they are “lucky” to have found. In this context, D’Elia [40] suggests a general listening concept for physicians to appreciate the entire possible spectrum of emotions that highlight doctors’ valuable roles as social figures.

Finally, interactions are also characterized by the many participants involved. Other physicians as well as family members can be added to the standard patient-physician interaction. They illustrate this linked network’s extent. Blöß et al. [41] verify these findings along the “diagnostic odyssey” of people suffering with rare diseases from the health expert’s perspective, and conclude that diagnostic procedures still need major improvements in the rare disease field, especially in classifying incredibly rare diseases. Alternatively, Dudding-Byth [42] describes the transfers and diagnostics processes that general practitioners face.

In a second step, we carved out the extent of “participation during the decision-making process.” Interviews suggest that rare diseases necessitate a broader extent of engagement, and consequently more time and effort. This is especially the case in times of diagnosis, as patients search for applicable therapy options and the initiation of medical treatment. People affected—either those who have lived with their diseases for a long time, and/or those who have chosen to settle with their diseases and take a more passive role—hand over their responsibilities to the doctor in a preferable “supervisory relationship.” Tofan et al. [43] describe this process in the agency theory context; in his eyes this is rational behavior, as one presumes physicians fully follow the Hippocratic tradition.

In contrast, reports also describe an active, engaged attitude within the healthcare system, in that patients may travel several hundred kilometers to find the right physician or specialized rare diseases center. The subsequent efforts are extensive, especially when young children are affected. Similarly, Dellve et al. [44] describe the high pressure parents face in the rare disease field.

Interviews confirm the particular relevance of “information exchange” in the rare disease field. The identified themes highlight the scarce professional healthcare knowledge on rare diseases, the ideal scope of health communications, as well as the people affected, who become “patient experts” or “lay experts” on their diseases. Literature often quotes this concept [26, 45], but this is also confirmed during this study. These interviews augment this concept and depict physicians’ difficult role, which shifts from a health information monopoly [46] to a new role as the sorter and structurer of available rare disease information.. Literature presents similar concerns from critics of the SDM model, who argue that most patients do not want to participate in such decisions, and revealing the uncertainties in medicine should be harmful. Further, while presenting all the potential risks and benefits across all treatment options is not feasible, the greatest concern is that increasing patient involvement in decision-making can lead to a greater demand for unnecessary, costly, or harmful medical procedures [47]. Therefore, the ultimate goal should be to quantify costs and benefits in a controlled health-economic setting.

Potentials exist for patients who enthusiastically collect data, either from the Internet or from many interactions with experts during their path through the healthcare system. However, this potential is still hindered at a communication level, as the people affected may be concerned with negative reactions in their interactions with professionals. Literature also often describes Internet-based health information searches. For example, McMullan [48] notes different access timeframes, and highlights the threat resulting from information sources of diverging quality [45, 49, 50].

Finally, the last item suggested by Charles et al. [6] is the decision-making agreement itself. This study’s interviews presented all forms of decision-making and agreement, and predominately described informed individual decision-making followed by paternalistic approaches. Although literature [7] suggests its increasing prominence, remarkably the SDM concept was rarely described. Further, SDM is difficult to identify, as the items overlap and are often difficult to grasp, as mutual acceptance and agreement can be stated at one point in time but may not hold [6]. Eliacin et al. [9] analyze patients’ definitions of SDM to discover that the understanding of the concept in practice is consistent with literature. However, study participants indicated that SDM is not limited to the models suggested in literature.

Paternalistic approaches were depicted as unavoidable—such as for example in emergency situations—. These findings have also been reported by Budych, Helms, and Schulz [51], although these authors do not provide a context in which these approaches were welcome. Baron, Reyher, and Stack [52] report positive outcomes from paternalistic approaches in a crisis situation. The patients in their study were treated paternalistically, and exhibited a higher responsiveness to suggestibility (*p* > .001), felt they could depend more on the physician, perceived him as warmer and more supportive (*p* > .01), and expressed fewer incidences of physiological distress compared with patients treated in an egalitarian manner.

Informed decision-making was also often described to highlight patients’ independence, and especially as they decide when to consult a healthcare professional and the extent to which patients consult their physician. Patients then decide whether to follow physicians’ decisions or change medical consultants. Thus, it is often assumed that patients prefer Internet-based health information, while concerns simultaneously exist that this would lead to extensive, costly healthcare [47].

In this context, the potential of the shared decision-making process shows that integrating both “lay expert” knowledge and efforts as well as professional knowledge can soften conflicts and strengthen the rare diseases network approach at its core. Well-conducted SDM enhanced reported satisfaction, understanding, and confidence in the decisions [53]. In this regard, strengthening the patient-physician relationship through SDM can potentially diminish within the field of rare diseases the highly relevant issue of doctor shopping. Others regard the process of decision-making in its entirety, and insinuate the importance of increased involvement of participants and the approachability of providers [560]. Finally, observational studies with patients suffering from hip and knee osteoarthritis showed they choose less expensive medical procedures when SDM was chosen as a decision-making tool resulting in a cost reduction of approximately 12 to 21% [54]. This leads to the assumption that cost-effectiveness can be further improved through the implementation of such concepts. Considering the potential, we suggest further health economic evaluations in the field of rare diseases to generate knowledge on the benefits of such approaches.

### The study’s significance

To our knowledge, the current study is unique in its approach: it complements Charles et al.’s [6] SDM model, and integrates perspectives from physicians and patients’ family members concerning this matter across a range of rare diseases. It also verifies findings from Budych, Helms, and Schultz [51], who also advocated for a conscious exchange of information in the rare disease field, although not within the context of a set framework. Moreover, they only focused on some rare diseases and the patient’s perspective. The underlying study covers all disease areas and integrates different perspectives to illustrate a broader picture and contribute to an advocated network approach at a micro-level in the healthcare system.

Patients’ perspectives at the macro-level are often systematically included by involving patient representatives. At the meso-level, it has been demonstrated that group decision-making tools can systematically integrate patient perspectives [21]. Thus, the question arises regarding which tool should be chosen to further implement SDM in practice. Literature suggests coaching programs or workshops [55], as well as decision boxes or tools led by nurses [11]. Scholl et al. [56] report the limited validity of SDM tools, and indicate why such concepts may not have been established in practice. Further, Elwyn et al. [5] describe criteria for a practical implementation to overcome these hurdles.

The patient participation concept has been integrated within Germany’s national plan for cancer care thus far, but has not yet become an integral part of the German rare disease national plan [20]. Following the model established by the national plan for cancerous diseases, patient perspectives can also become systematically integrated at the micro-health and economic levels. This can be accomplished by integrating SDM within various concepts for further development in the field, therefore ensuring and controlling its long-term application.

### Assumptions and limitations

This study was conducted using qualitative interviews, and participants were encouraged to report their experiences with the healthcare system during their disease diagnosis and management, assuming that this reflects the actual subjective relevance in the decision-making process. The statements’ validity have been verified by triangulation. Therefore, only a limited number of patients, family members, and physicians could be interviewed, but the number of participants was sufficient as a base knowledge generation was achieved. The qualitative design contributes to theory generation by gathering relevant items and avoiding absolute numerical statements; results must be verified through a quantitative study to make further projections and/or obtain evidence.

Shared-decision making was not addressed as such within the interview guide. Participants were not directly asked about SDM, but rather indirectly about their experiences within healthcare systems and health management during the information-gathering process. This approach bears some risk, in that some interviews may involve situations in which SDM did not occur. One advantage of this approach is its identification of the actual perceptions of SDM, decreasing potential biases. By avoiding the SDM concept but focusing on informed health behavior, participants cannot be cued regarding how to answer the question to appease the researchers [57]. However, this also bears the risk that the concept may not impose itself in its full extensity, as it was avoided to pinpoint participants towards the concept, by directly asking for it.

The interview study was conducted in 2014, but several new rare disease centers have been subsequently established, and a national rare disease plan has been pushed toward implementation. However, the general structure of this matter has not changed to impact the German healthcare system.

Besides, we did not interview the treating physicians of the included patients and or their family members due to organizational restraints. The patients interviewed in the current study were predominantly female; in this context, Wyatt et al. [58] reveal that no gender specifications exist in decisional conflicts, patients’ satisfaction with the clinical encounter, or patients’ engagement at the point of encounter. Only an increased concordance between decisions and actions were described in encounters with female clinicians, while male patients demonstrated an increased concordance in the decision aid arm compared to the control arm (*p* = 0.05). Further, women more actively manage their healthcare status; for example, woman search more often for Internet-based health information [59].

## Conclusions

To the best of our knowledge, this study is the first to provide unique insights on the decision-making practice and SDM’s current relevance within the rare disease field. While SDM is increasingly present, the reported processes still lack many aspects of the decision-making process in this area. Further, a shared decision-making agreement was more rarely reported; the patient-physician relationship was characterized by a distorted trust-building process; and such characteristics of rare diseases as genetic origins, severity, and chronic course may lead to patients’ high dependencies on their physicians. Patients may also often suffer from stigmatization as stimulants. Although the physicians in our study noted that they would need further time to analyze rare disease cases, participation was comparably pronounced regarding their patient-side engagement. Political health efforts should strive toward these efforts and promote diminishment as strengths vanish during the odyssey through healthcare systems. The particular role of “expert patients” or “lay experts” in the rare disease field has again unfolded, and potential especially surfaces regarding the integration of the information gathered during the decision-making process.

The aforementioned efficiency potentials can be triggered through a further integration of shared decision-making, facilitating diagnostics and disease management. It is also noteworthy that the integration of shared decision-making in the rare disease field not only requires strengthening patients’ positioning, but also the positioning of physicians. These potentials can be triggered by implementing further SDM processes within the rare disease field, for example, through integrating participating decision-making concepts within rare disease national plans, as has already been accomplished in the cancerous disease field. This can provide an opportunity to reinforce a crucially relevant networking approach, strengthened by rare disease centers and guides at its core, on a micro-level, and within patient-physician interactions. Further research can quantify this potential and examine the health-economic impacts of shared decision-making on overall healthcare spending.

## Data Availability

The data that support this study’s findings are available from the Center for Health Economics Research Hannover (CHERH), but restrictions apply to the data availability, which were used under license for the current study, and thus are not publicly available. However, data are available from the authors upon reasonable request and with the study participants’ permission.

## Appendix 1

**Table 5:** Characteristics of patients, family members, and physicians (*N* = 107)

Parameters	Patients (*n* = 55)		Family members (*n* = 13)		Physicians (*n* = 33)	
Sex						
Male		18		5		23
Female		37		8		10
Age						
Average		52		44		48
Maximum		85		60		68
Minimum		18		28		31
Civil status						
No specification		–		1		–
Married/cohabiting		34		9		–
Single		11		1		–
Divorced/Separated		7		1		–
Widowed		3		1		–
Educational qualification						
Technical collage/ university degree		14		5		–
Abitur		8		5		–
Advanced technical college degree		5		0		–
Secondary education		18		1		–
Secondary modern school qualification		10		2		–
Members of the household						–
Average		2,09		3,58		–
Maximum		5		5		–
Minimum		1		1		–
Age at diagnosis						
Average		37		8		–
Maximum		74		39		–
Minimum		0		0		–
Disease severity						
No specification		–		0		–
Low		5		3		–
Medium		26		2		–
Severe		21		7		–
Profession						
Employed		22		9		–
Unemployable		12		1		–
Pensioner		18		–		–
Student/Scholar		2		–		–
Homemaker	–			2		–
Special circumstances		1		–		–
Medical rare disease experience / RD guide		–		–		6
Regional aspects						
Rural		–		–		3
Urban		–		–		7
Metropolitan		–		–		8
Practice form						
Single practice		–		–		7
Group practice		–		–		11
Clinic level						
Basic		–		–		4
Regular		–		–		0
Specialist		–		–		1
Maximum medical care		–		–		4

## Appendix 2

### Interview guide

Section I: Disease.

This is for patients and their relatives when a diagnosis can be consciously discerned.

Question 1:

- I would like you to remember the onset of your / your relative’s disease. What kind of changes did you perceive?

- Optional subquestion(s):

o Can you remember specific events?

o What kind of changes became noticeable within your body?

o To what extent did you perceive changes within your social environment?

o If the disease was diagnosed at birth, continue with Section II.

Question 2:

- How did the diagnosis occur?

Question 3:

- What happened after the diagnosis?

- Optional subquestion:

o How did the illness progress from there?

Question 4:

When you place yourself back in the situation, what did you feel?

Section II: Disease.

This is for patients only, when a diagnosis was not consciously discerned.

Question 1:

- I would like you to tell me about your disease and how your life changed. You may take all the time necessary for your answer.

- Optional subquestion:

o Describe the course of the disease. (For example: Were there any acute phases?)

Question 2:

- How did the illness manifest in your everyday life?

- Optional subquestion:

o Have you experienced any limitations, and if so, what kind?

Question 3:

- Some people have the desire to become informed themselves about disease. How is it with you?

- Optional subquestion:

o Can you describe a situation in which you desired to acquire more information about your disease and its management?

Section III: The search and need for information.

Question 1:

- Describe searching for information about the handling of your disease.

- Optional subquestion(s):

o Please try to remember what kind of information you searched for.

o Where did you find the information?

o How satisfied were you with your search results?

Question 2:

- Were there any events before or after which you more intensely searched for information?

- Optional subquestion(s):

o How did you proceed with your search?

Question 3:

- To what extent were there situations in which you could easily access information?

- Optional subquestion(s):

o What types of information were these?

Question 4:

- To what extent were there situations in which you strove to find information, but could not find it?

- Optional subquestion(s):

o What types of information were these?

o What kind of information would you wish for?

o What kind of information do you think you will need in the future?

Question 5:

- What moments were significant during your search?

- Optional subquestion(s):

o Was there a point in time at which you felt you had achieved a breakthrough?

Section IV: Media or respective information access points.

Question 1:

- Please consider the many possibilities through which one can presently communicate with the help of modern or classic media. When you consider your own situation, what possibilities did you use during your own search?

- Optional subquestion(s):

- What do you comprehend as the communication possibilities for modern and classic media, respectively?

Question 2:

- In what ways would you like to access information?

- Optional subquestion(s):

o Would you also like to access information using your mobile device or smartphone?

o What is your opinion of accessing information through social media, such as Twitter or Facebook?

o For example, we have considered integrating a helpline as part of a national information website. What is your opinion regarding the possibility of a helpline? How do you envision such a helpline?

Section V: Windup.

Question:

- Are there any other topics that you would like to address?

- Optional subquestion(s):

o Are there any other important aspects that we have not yet addressed?

## Abbreviations

SDM: Shared decision-making
ZIPSE: Zentrale Informationsportal über seltene Erkrankungen
